# Riga – Plate flow of γ Al_2_O_3_-water/ethylene glycol with effective Prandtl number impacts

**DOI:** 10.1016/j.heliyon.2019.e01651

**Published:** 2019-05-20

**Authors:** N. Vishnu Ganesh, Qasem M. Al-Mdallal, Sara Al Fahel, Shymaa Dadoa

**Affiliations:** a Department of Mathematics, Ramakrishna Mission Vivekananda College, Mylapore, Chennai, 600004, Tamil Nadu, India; b Department of Mathematical Sciences, United Arab Emirates University, P.O. Box 15551, Al Ain, Abu Dhabi, United Arab Emirates

**Keywords:** Applied mathematics, Computational mathematics, Mechanical engineering

## Abstract

In many industrial processes, the cooling process can be improved by varying the flow geometry or changing the additives in the working fluid. The present work concentrates on the flow of γ Al_2_O_3_ –Water/Ethylene Glycol over a Gailitis and Lielausis device with an effective Prandtl number for the first time. The thermal transport aspects of electro-MHD boundary layer flow of γ Al_2_O_3_ nanofluids over a stretchable Riga plate are studied in two dimensions. The wall parallel Lorentz force is produced due to an external electric field by Riga plate to control the nanofluid flow. Mathematical models are developed with an effective Prandtl number. The no-slip and the prescribed surface temperature boundary conditions are assumed. Results are discussed using numerical results obtained by fourth order RK method with shooting technique. Special case analytical solutions are presented for both momentum and energy equations. The increasing behaviour in velocity profile and decreasing behaviours in temperature, skin friction and Nusselt number are observed with increasing modified Hartmann number. The higher modified Hartmann number leads to a sudden enhancement in the velocity profile of the nanofluid in the presence of effective Pr near the riga plate wall.

## Nomenclature

***B***: non-dimensional number
***M***: magnetization of the permanent magnets
***T***: temperature of the nanofluid
***T***_***w***_: temperature of the nanofluid on the wall
$T_{\infty }$: ambient temperature
***Pr***_***nf***_: Prandtl number of the nanofluid
***Pr***_***f***_: Prandtl number of the base fluid
12Rex1/2Cf: local skin friction coefficient
Rex−1/2Nux: reduced Nusselt number
***Z***: modified Hartmann number
**c**: width of electrodes and magnets
***g***: acceleration due to gravity
***j***_***0***_: applied current density
***k***_***nf***_: thermal conductivity of the nanofluid
***k***_***f***_: thermal conductivity of the base fluid
***k***_***s***_: thermal conductivity of the nanoparticles
***u,v***: velocity components in *x* and *y* directions, respectively
***u***_***w***_: stretching velocity
***ϕ***: nanoparticle volume fraction
$\rho_{nf}$: effective density of the nanofluid
$\rho_{f}$: density of the base fluid
$\rho_{s}$: density of the nanoparticles
$\mu_{nf}$: effective dynamic viscosity of the nanofluid
$\mu_{f}$: dynamic viscosity of the base fluid
***η***: space variable

## 1 Introduction

Magnetohydrodynamics is a branch of modern theory of fluid dynamics that characterizes the electrohydromagnetic processes arising in electric conducting flows under the influence of magnetic field. In classical MHD, the flow of highly electric conducting fluids could be dominated by an external magnetic field. But, the applied external magnetic field produces very small amount of current in weakly electric conducting fluids (e.g. sea water). The efficient flow control can be achieved only by applying the Lorentz force in wall parallel direction. Gailitis and Lielausis [1] designed a device called Riga-plate to produce the Lorentz force in the direction which is parallel to the wall. Riga plate is an electromagnetic actuator which includes span wise aligned array of alternating electrodes and permanent magnets, mounted on a plane surface [2, 3]. It can be utilized to reduce the friction force and pressure drag of submarines by avoiding the boundary layer separation and decrease the production of turbulence. Tsinober and Shtern [4] reported that the impacts of applying the Lorentz forces in wall-parallel direction are useful to increase the stability of Blasius flow over a Riga plate. The effects of this type of Lorentz force on the boundary layer flow of viscous fluid are investigate in recent years [5, 6].

In many of the industrial applications, the heat transfer enhancement methods are needed for high performance cooling or heating. But these methods are limited by the restriction of the low thermal conductivity of convectional heat transfer liquids like oil, water, ethylene glycol etc. Choi [7] introduced an advanced fluid called nanofluid and suggested to replace the conventional fluids with theses advanced fluids. The main idea is to combine the conventional fluids and nanosized solid particles of high thermal conductivity [8, 9, 10, 11, 12, 13, 14, 15, 16]. Nanofluids have better wetting. dispersion and separation properties on the surfaces such as stretching plate, Riga plate and surfaces with variable thickness. The suitable nanoparticle additives in the working fluid have much influence in the enhancement of thermal conductivity of base working fluid. Such investigations have significance in thermal treatment of cancer, aerospace, micro electronics and medical applications. The recent developments in the nanofluid theory, modelling and applications can be found in the articles of Mahian et al . 17, 18 . Akbarzadeh [19] studied the MHD nanofluid flow between a porous layer in the presence of internal heat generation. Golafshan and Rahimi [20] investigated the effect of radiation on the third grade nanofluid over a stretching sheet with MHD effects. Freidoonimehr and Rahimi [21] examined the Brownian motion and slip effects on the three dimensional nanofluid flow. Khan et al. [22] addressed the impacts of nonlinear radiation on the flow of cross nanofluid. The development in the nanofluid flow with various physical aspects has been analysed in the papers 23, 24, 25, 26, 27. Recently, the γ Al_2_O_3_ nanofluids are being studied by the many experimental and theoretical, researchers due to its variety of cooling applications. Maiga et al. 28, 29, 30 studied the heat transfer characteristics of γ Al_2_O_3_ nanofluids in heated tubes. Pop et al. [31] have made an analysis of laminar-to-turbulent threshold with γ Al_2_O_3_ nanofluids. Farajollahi et al. [32] reported the heat transfer characteristics of γ-Al_2_O_3_/water and TiO_2_/water in a shell and tube with turbulent flow condition. Sow et al. [33] have done an experimental study on the freezing point of γ- Al_2_O_3_ water nanofluid. Beiki et al [34] considered the forced laminar flow of γ-Al_2_O_3_/electrolyte nanofluid in a circular tube. Esmaeilzadeh et al. [35] studied the heat transfer and friction factor of γ-Al_2_O_3_/water through circular tube with twisted tape inserts with different thicknesses. Abdul et al. [36] used γ Al_2_O_3_ nano-fluid to analyse the effect of operating parameters on the gravity assisted heat pipe. Bayomy et al [37] have done a numerical and experimental work on the flow of γ-Al_2_O_3_–water nanofluid through aluminum foam heat sink. Moghaieb et al [38] utilized γAl_2_O_3_/Water nanofluids as a engine coolant in their study. Vishnu Ganesh and his co-authors 39, 40, 41, 42 studied the boundary layer flow of γAl_2_O_3_ nanofluids with various physical effects. Ahmad et al. [43] studied the strong suction effects on Riga plate nanofluid flow region. Hayat et al. [44] investigated the characteristics of Riga plate flow of nanofluid in which the plate was convectively heated. The slip effects on Riga plate flow of nanofluid was analysed by Ayub et al [45]. Recently, Ahmad et al. [46] studied the vertical Riga plate flow of nanofluid.

Motivated by the above works, an attempt has been taken to study the γ Al_2_O_3_-Water/Ethylene glycol nanofluid flow over a stretchable Riga plate with the impacts of effective Prandtl number and electro-magnetohydrodynamics. The related mathematical formulation has been done with an effective Prandtl number. The Grinberg term [3] has been used to model the electro MHD flow of nanofluids. Numerical solutions are carried out by fourth order RK method and special case analytical solutions are presented.

## 2 Methodology

### 2.1 Problem formulation

**Fig. 1:**
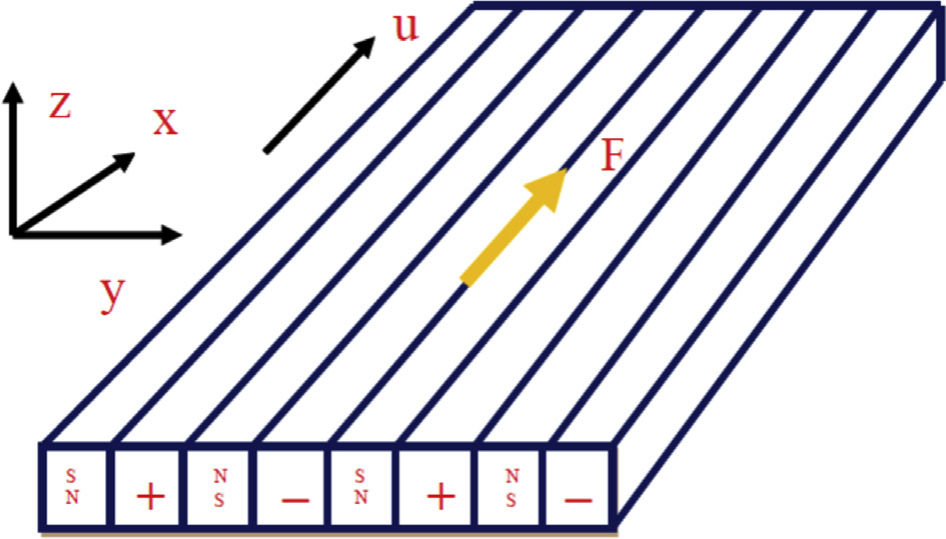
Sketch of riga plate.

**Fig. 2:**
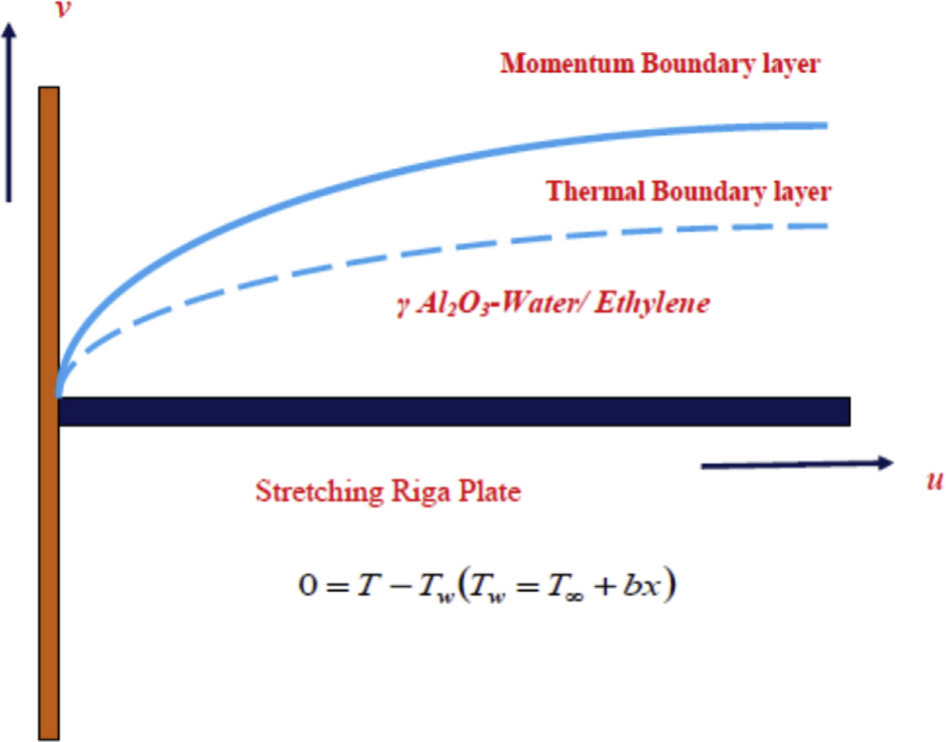
Schematic of physical model.

Two dimensional, steady, electro MHD flow of γ Al_2_O_3_-Water/Ethylene glycol nanofluid over a stretching Riga plate with stretching velocity $u_{w}=a\;x$ is considered (See Figs. 1 and 2). The prescribed surface temperature at the Riga plate is $T_{w}=T_{\infty }+b\;x$. Where $T_{\infty }$ is the ambient temperature and a and *b* are constants. It is assumed that no-slip condition and thermally equilibrium state between γ Al_2_O_3_ nanoparticles and base fluids. With the above assumptions, the governing equations of the problem are as follows

(1) $$\frac{\partial u}{\partial x}=-\frac{\partial v}{\partial y},$$

(2) $$\frac{\partial u}{\partial x}u+\frac{\partial u}{\partial y}v-\frac{\mu_{nf}}{\rho_{nf}}\frac{\partial^{2}u}{\partial y^{2}}-\frac{\pi j_{0}M}{8\rho_{nf}}e^{\left(\frac{-\pi }{c}y\right)}=0$$

(3) $$\frac{\partial T}{\partial x}u+\frac{\partial T}{\partial y}v-\frac{k_{nf}}{{\left(\rho C_{p}\right)}_{nf}}\frac{\partial^{2}T}{\partial y^{2}}=0.$$

The no-slip and the PST boundary conditions are

(4) $$0=u-u_{w},0=v\text{,}\;0=T-T_{w}\left(T_{w}=T_{\infty }+bx\right)\;\text{at}\;y=0\text{,}\;u\to 0\text{,}\;T\to T_{\infty }\;\text{as}\;y\to \infty \text{,}$$

where *u* and *v* are the components of velocity along the *x* and *y* directions*,* respectively, *M=M*_*0*_ *x* is the magnetization of the permanent magnets, *j*_*0*_ is the applied current density and *c* is the width of electrodes and magnets [47].

The effective dynamic density ($\rho_{nf}$) and the heat capacitance (${\left(\rho C_{p}\right)}_{nf}$) are given by

(5) $$\rho_{nf}=\left(1-\varphi \right)\rho_{f}+\varphi \rho_{s}\text{,}\;{\left(\rho C_{p}\right)}_{nf}=\left(1-\varphi \right){\left(\rho C_{p}\right)}_{f}+\varphi {\left(\rho C_{p}\right)}_{s}\text{,}$$

where ϕ is the solid volume fraction of nanofluid.

The dynamic viscosity of the nanofluid is defined as

(6) $$\frac{\mu_{nf}}{\mu_{f}}=123\varphi^{2}+7.3\varphi +1\text{,}\;\left(\text{for}\;\gamma \;{Al}_{2}O_{3-}\;Water\right)\text{,}$$

(7) $$\frac{\mu_{nf}}{\mu_{f}}=306\varphi^{2}-0.19\varphi +1\text{,}\;\left(\text{for}\;\gamma \;{Al}_{2}O_{3-}\;\mathrm{Ethylene}\;\mathrm{glycol}\right)\text{.}$$

The effective thermal conductivity of the nanofluid is given by

(8) $$\frac{k_{nf}}{k_{f}}=4.97\varphi^{2}+2.72\varphi +1\text{,}\;\left(\text{for}\;\gamma \;{\mathrm{Al}}_{2}O_{3-}\;\mathrm{Water}\right)\text{,}$$

(9) $$\frac{k_{nf}}{k_{f}}=28.905\varphi^{2}+2.8273\varphi +1\text{,}\;\left(\text{for}\;\gamma \;{\mathrm{Al}}_{2}O_{3-}\;\mathrm{Ethylene}\;\mathrm{glycol}\right)\text{.}$$

The effective Prandtl number of the nanofluid is given by

(10) $$\frac{{\mathrm{Pr}}_{nf}}{{\mathrm{Pr}}_{f}}=82.1\phi^{2}+3.9\phi +1\text{,}\;\left(\text{for}\;\gamma \;{\mathrm{Al}}_{2}O_{3-}\;\mathrm{Water}\right)\text{,}$$

(11) $$\frac{{\mathrm{Pr}}_{nf}}{{\mathrm{Pr}}_{f}}=254.3\varphi^{2}-3\varphi +1\text{,}\;\left(\text{for}\;\gamma \;{\mathrm{Al}}_{2}O_{3-}\mathrm{Ethylene}\;\mathrm{glycol}\right)\text{.}$$

Eq. (5) is the common correlation used to calculate the $\rho_{nf}$ and ${\left(\rho C_{p}\right)}_{nf}$. Eqs. (6), (7), (8), and (9) are the dynamic viscosity and the effective thermal conductivity of *γ Al*_*2*_*O*_*3*_ nanofluids that have been obtained by performing a curve fitting (least square) of some experimental data [28, 29, 30, 48, 49, 50]. Eqs. (8) and (9) are obtained from Hamilton and Crosser model [51]. Eqs. (10) and (11) are the effective Prandtl number models which are obtained by a curve fitting using regression laws [31, 40].

By using the following relations

(12) $$\eta -\sqrt{\frac{a}{v_{f}}}y=0\text{,}\;u-axf'\left(\eta \right)=0\text{,}\;v+{\left(av_{f}\right)}^{1/2}f\left(\eta \right)=\text{0}\;\text{and}\;\theta =\frac{T-T_{\infty }}{T_{w}-T_{\infty }}\text{,}$$

Eqs. (2) and (3) are transformed to non-dimensional form as follow:

(13) $$f'''=-\frac{\left(1-\varphi +\varphi \left(\frac{\rho_{s}}{\rho_{f}}\right)\right)\left(ff''-f{'}^{2}\right)}{\left(123\varphi^{2}+7.3\varphi +1\right)}\;-\;\frac{Ze^{-B\eta }}{\left(123\varphi^{2}+7.3\varphi +1\right)}\left(\text{for}\;\gamma \;{\mathrm{Al}}_{2}O_{3-}\;\mathrm{Water}\right)\text{,}$$

(14) $$f'''=-\frac{\left(1-\varphi +\varphi \left(\frac{\rho_{s}}{\rho_{f}}\right)\right)\left(ff''-f{'}^{2}\right)}{\left(306\varphi^{2}-0.19\varphi +1\right)}-\frac{Ze^{-B\eta }}{\left(306\varphi^{2}-0.19\varphi +1\right)}\left(\text{for}\;\gamma \;{\mathrm{Al}}_{2}O_{3-}\;\mathrm{Ethylene}\;\mathrm{glycol}\right)$$

(15) $$\theta ''=-\frac{{\mathrm{Pr}}_{f}\left(1-\varphi +\varphi \left(\frac{\rho_{s}}{\rho_{f}}\right)\right)\left(82.1\varphi^{2}+3.9\varphi +1\right)}{123\varphi^{2}+7.3\varphi +1}\left(f\theta '-\theta f'\right)\left(\text{for}\;\gamma \;{\mathrm{Al}}_{2}O_{3-}\;\mathrm{Water}\right)\text{,}$$

(16) $$\theta ''=-\frac{{\mathrm{Pr}}_{f}\left(1-\varphi +\varphi \left(\frac{\rho_{s}}{\rho_{f}}\right)\right)\left(254.3\varphi^{2}-3\varphi +1\right)}{306\varphi^{2}-0.19\varphi +1}\left(f\theta '-\theta f'\right)\;\left(\text{for}\;\gamma \;{\mathrm{Al}}_{2}O_{3-}\;\mathrm{Ethylene}\;\mathrm{glycol}\right)\text{.}$$

The corresponding non-dimensional Riga plate flow boundary conditions are

(17) 0=f(0),1=f'(0),0=f'(∞),1=θ(0)and0=θ(∞).

Where B is a non-dimensional number, $Z=\left(\frac{\pi \;j_{0}\;M_{0}}{8\;\rho_{f\;}\;a{\;}^{2}}\right)$ is the modified Hartmann number, Pr is the Prandtl number and ϕ is volume fraction of nanoparticles.

One can observe that if modified Hartmann number Z = 0, the present problem reduces to the stretching sheet problem of nanofluid. An exact solution to the momentum Eqs. (13) and (14) with Z = 0 is obtained as [40].

$$f\left(\eta \right)=\frac{1-e^{-\alpha \eta }}{\alpha }$$

Where

$$\alpha =\sqrt{\left(1-\varphi +\varphi \left(\frac{\rho_{s}}{\rho_{f}}\right)\right){\left(123\varphi^{2}+7.3\varphi +1\right)}^{-1}}\left(\text{for}\;\gamma \;{\mathrm{Al}}_{2}O_{3-}\mathrm{Water}\right)\text{,}$$

$$\alpha =\sqrt{\left(1-\varphi +\varphi \left(\frac{\rho_{s}}{\rho_{f}}\right)\right){\left(306\varphi^{2}-0.19\varphi +1\right)}^{-1}}\left(\text{for}\;\gamma \;{\mathrm{Al}}_{2}O_{3-}\mathrm{Ethylene}\;\mathrm{glycol}\right)\text{.}$$

The hypergeometric function solution for the energy Eqs. (8) and (9) along with Eq. (10) with Z = 0 is obtained as [40] (see Table 1).

$$\theta \left(\eta \right)=e^{-\left(A\;\alpha^{-2}\right)\alpha \;\eta }\left[M\left(E\;\alpha^{-2}-1,\;1+\;E\;\alpha^{-2},\;-E\alpha^{-2}\;e^{-\alpha \;\eta }\right)\;\;M{\left(\alpha -1,\;1+\;\alpha ,\;-E\alpha^{-2}\;\right)}^{-1}\right]$$

$$\text{where}\;E=\frac{{\mathrm{Pr}}_{f}\left(1-\varphi +\varphi \left(\frac{\rho_{s}}{\rho_{f}}\right)\right)\left(82.1\varphi^{2}+3.9\varphi +1\right)}{123\varphi^{2}+7.3\varphi +1}\left(\text{for}\;\gamma \;{\mathrm{Al}}_{2}O_{3-}\mathrm{Water}\right)\;\text{and}$$

$$E=\frac{{\mathrm{Pr}}_{f}\left(1-\varphi +\varphi \left(\frac{\rho_{s}}{\rho_{f}}\right)\right)\left(254.3\varphi^{2}-3\varphi +1\right)}{306\varphi^{2}-0.19\varphi +1}\left(\text{for}\;\gamma \;{\mathrm{Al}}_{2}O_{3-}\;\mathrm{Ethylene}\;\mathrm{glycol}\right)\text{.}$$

**Table 1:** Thermo physical properties of water, ethylene glycol and alumina.

	ρ (kg/m^3^)	C_p_ (J/kg K)	k (W/m K)	β x 10^−5^ (K^−1^)	μ (kg/m.s)	Pr
Pure water (H_2_O)	998.3	4182	0.60	20.06	0.0009985653	6.96
Ethylene glycol (C_2_H_6_O_2_)	1116.6	2382	0.249	65	0.021324937	204
Alumina (Al_2_O_3_)	3970	765	40	0.85		-

The local skin friction coefficient Rex1/2Cf and the reduced Nusselt number Rex−1/2Nux are derived and given in Table 2 for γ Al_2_O_3_ –Water/Ethylene Glycol nanofluids.

**Table 2:** The local skin friction coefficient and the reduced Nusselt number for γ Al_2_O_3_ nanofluids.

	γ Al_2_O_3_-Water	γ Al_2_O_3_ Ethylene Glycol
Local skin friction coefficient	$-\left(123\varphi^{2}+7.3\varphi +1\right)f''\left(0\right)$	$-\left(306\varphi^{2}-0.19\varphi +1\right)f''\left(0\right)$
Reduced Nusselt number Rex−1/2Nux	$-\left(4.97\varphi^{2}+2.72\varphi +1\right)\theta '\left(0\right)$	$-\left(28.905\varphi^{2}+2.8273\varphi +1\right)\theta '\left(0\right)$

### 2.2 Numerical procedure

The transformed Eqs. (13), (14), (15), and (16) and the Riga plate flow BC's in (17) can be written in the following IVP form

(18) $$\left[\begin{array}{c} y_{1}^{'}\\ y_{2}^{'}\\ y_{3}^{'}\\ \begin{array}{c} y_{4}^{'}\\ y_{5}^{'} \end{array} \end{array}\right]=\left[\begin{array}{c} y_{2}\\ y_{3}\\ C\left(y_{2}^{2}-y_{1}y_{3}\right)-DZe^{-B\eta }\\ \begin{array}{c} y_{5}^{'}\\ E\left(y_{4}y_{2}-y_{1}y_{5}\right) \end{array} \end{array}\right]$$

(19) $$\left[\begin{array}{c} y_{1}^{'}\\ y_{2}^{'}\\ y_{3}^{'}\\ \begin{array}{c} y_{4}^{'}\\ y_{5}^{'} \end{array} \end{array}\right]=\left[\begin{array}{c} 0\\ 1\\ g_{1}\\ \begin{array}{c} 1\\ g_{2} \end{array} \end{array}\right]$$

$$\text{Where}\;C=\frac{\left(1-\varphi +\varphi \left(\frac{\rho_{s}}{\rho_{f}}\right)\right)}{\left(123\varphi^{2}+7.3\varphi +1\right)}\;\text{and}\;D=\frac{1}{\left(123\varphi^{2}+7.3\varphi +1\right)}\left(\text{for}\;\gamma \;{\mathrm{Al}}_{2}O_{3-}\mathrm{Water}\right)\text{.}$$

and

$$C=\frac{\left(1-\varphi +\varphi \left(\frac{\rho_{s}}{\rho_{f}}\right)\right)}{\left(306\varphi^{2}-0.19\varphi +1\right)}\;\text{and}\;D=\frac{1}{\left(306\varphi^{2}-0.19\varphi +1\right)}\;\left(\text{for}\;\gamma \;{\mathrm{Al}}_{2}O_{3-}\;\mathrm{Ethylene}\;\mathrm{glycol}\right)\text{.}$$

Eq. (18) and the initial conditions in (19) are solved using R–K integration technique along with shooting method. For the numerical computations, a convergence criterion of 10^−6^ has been used.

## 3 Results and discussion

Numerical results for velocity and temperature profiles are obtained by fourth order RK method with shooting techniques. To analyse the impacts of various pertinent parameters which involved in the problem are discussed via graphical illustrations. The verification of current numerical code has been done by comparing the reduced Nusselt number values with Isak [52] in the absence of modified Hartmann number and nanoparticle volume fraction. The comparisons of these values are in good agreement (Table 3.)

**Table 3:** Comparison results of -θ′(0) in the case of pure fluid and also in the absence of modified Hartmann number.

**Pr**	Comparison results of -θ′(0)	
	Present results	**Isak**[52]
**0.72**	0.808631	0.8086
**1.0**	1.000000	1.00000
**3.0**	1.923682	1.9237

**Fig. 3:**
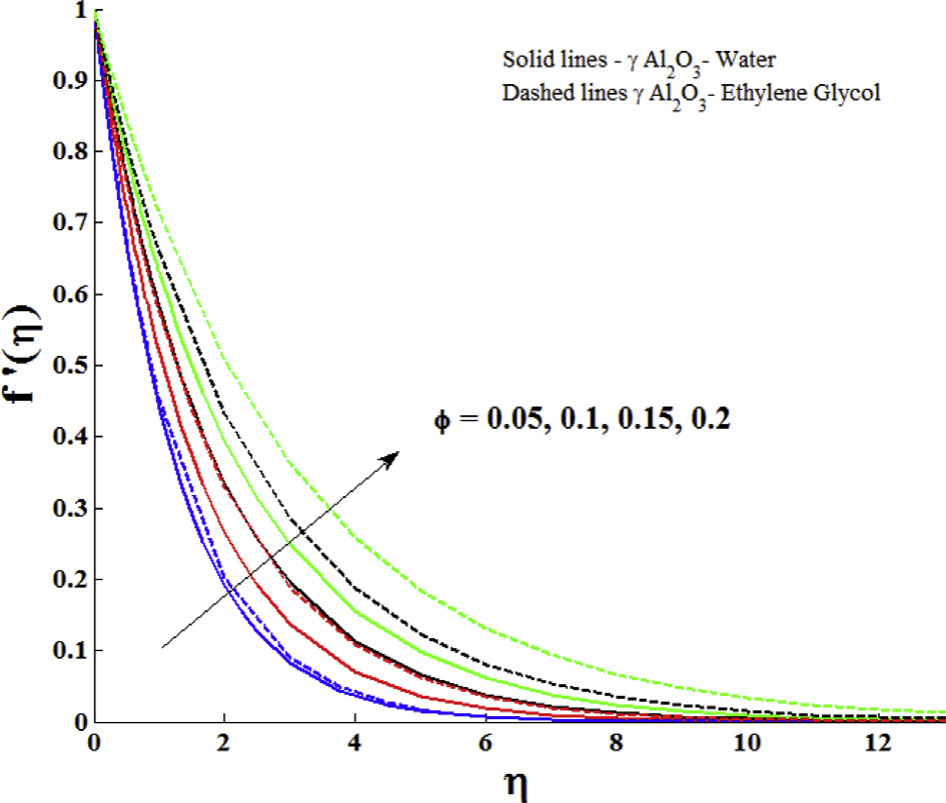
Impact of nanoparticle volume fraction (ϕ) on velocity profile with Z = 2.0.

The impacts of nanoparticle volume fraction ϕ of γ Al_2_O_3_ nanoparticles on the velocity profile with water and ethylene glycol as base fluids is described in Fig. 3. The velocity profile enhances with nanoparticle volume fraction of γ Al_2_O_3_ nanoparticles. The γ Al_2_O_3_ nanoparticles with same nanoparticle volume fraction show variations in velocity profile with different base fluids. On comparing the velocity profile of γ Al_2_O_3_- Water and γ Al_2_O_3_- ethylene glycol, it can be observe that the γ Al_2_O_3_- ethylene glycol has larger velocity.

**Fig. 4:**
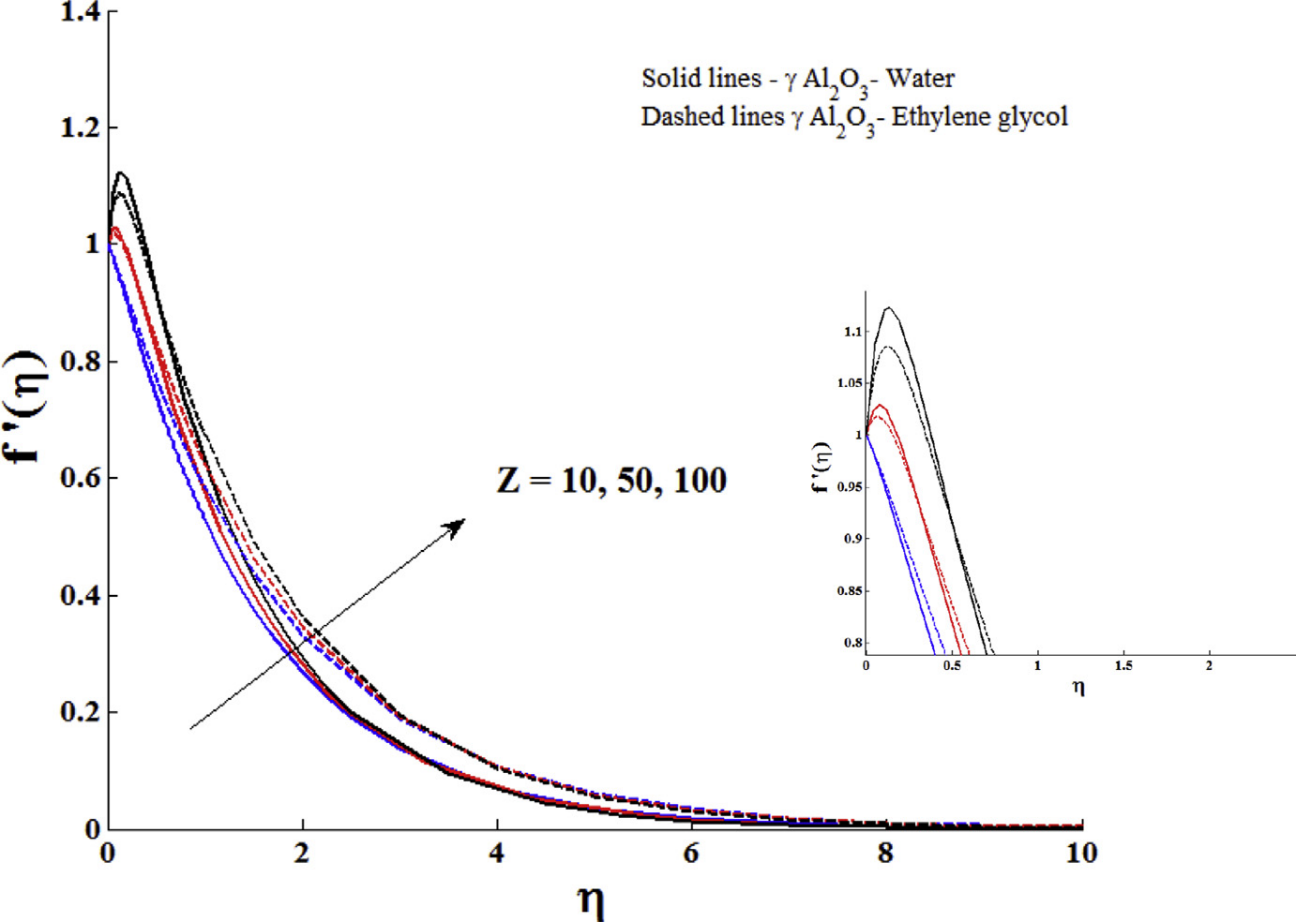
Impact of modified Hartmann number on velocity profile with ϕ = 0.1.

Characteristics of the modified Hartmann number Z on the velocity profile of the nanofluid are exposed in Fig. 4. An increasing behaviour of velocity profile due to the increase of modified Hartmann number has been seen in this figure. In fact, the larger values of this parameter lead to enhance the external electric field. This enhancement in external electric field leads to the production of wall parallel Lorentz force which slowing down the growth of the momentum boundary layer. Closely examining the figure, it is noted that the velocity suddenly rises near the plate with larger modified Hartmann number.

**Fig. 5:**
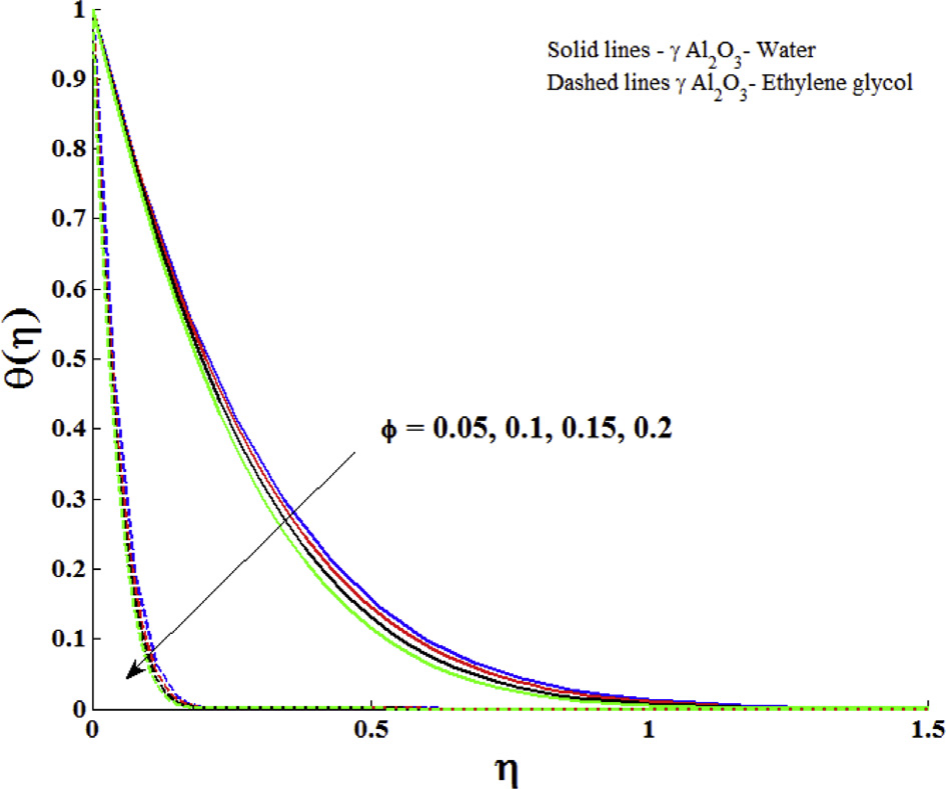
Impact of nanoparticle volume fraction (ϕ) on temperature profile with Z = 2.0, Pr = 6.96 (γ Al_2_O_3_- Water) and Pr = 204 (γ Al_2_O_3_- Ethylene glycol).

**Fig. 6:**
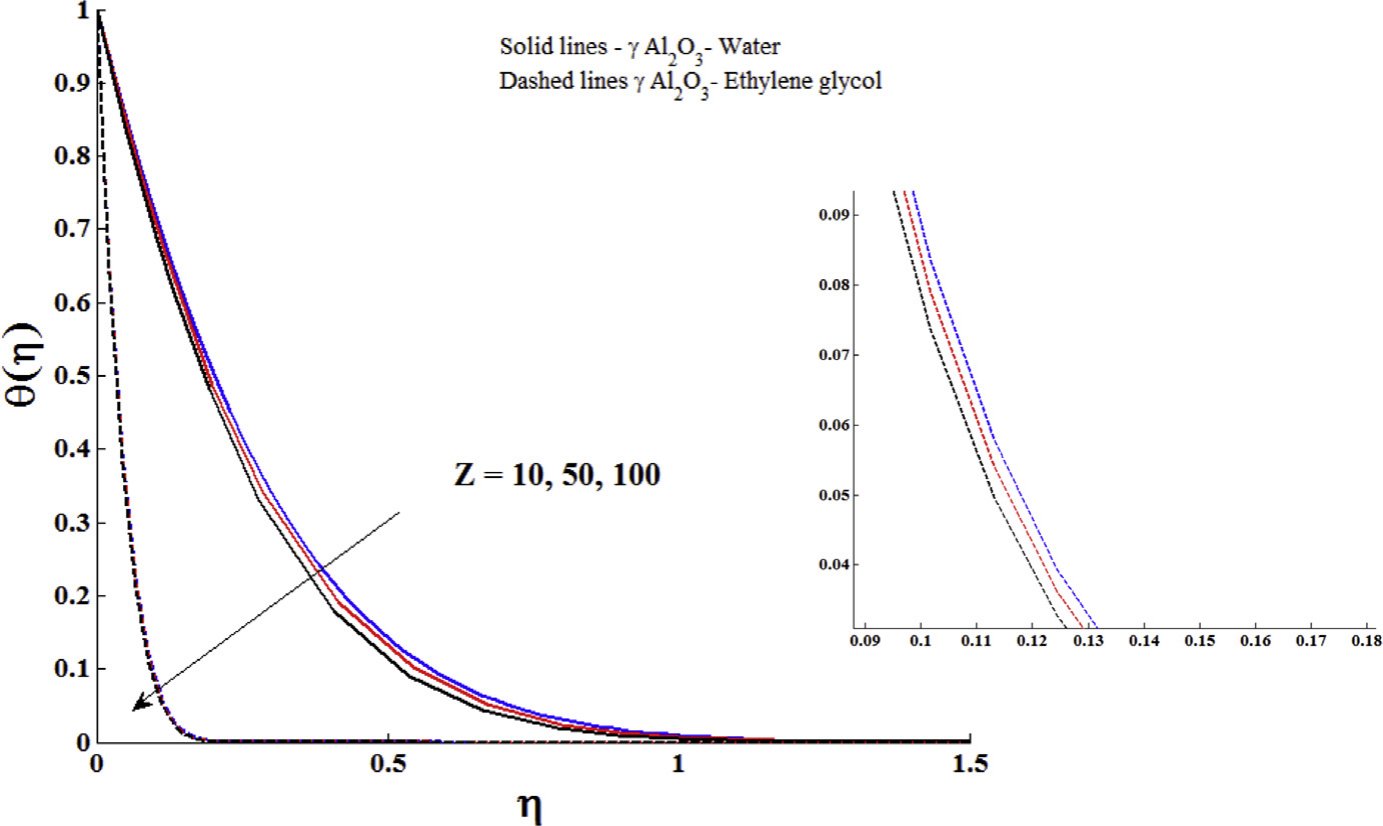
Impact of modified Hartmann number on temperature profile with ϕ = 0.1, Pr = 6.96 (γ Al_2_O_3_- Water) and Pr = 204 (γ Al_2_O_3_- Ethylene glycol).

Fig. 5 is prepared to show the influences of ϕ on the temperature profile of γ Al_2_O_3_ nanofluids. It is clear that the temperature profile is a decreasing function of nanoparticle volume fraction. Experimental studies have shown that the γ Al_2_O_3_ nanofluids are used for the cooling purposes [33, 34, 35, 36, 37, 38]. Thus the present theoretical result revealed that the same behaviour of γ Al_2_O_3_ nanofluids. On comparing the thermal boundary layers of γ Al_2_O_3_ -Water and γ Al_2_O_3_ - Ethylene glycol, it is observed that the thermal boundary layer of γ Al_2_O_3_ - Ethylene glycol is thinner than γ Al_2_O_3_ –Water. This is due to the fact that the value of k is higher for water than ethylene glycol. Fig. 6 portrays the influences Z on the temperature profile of γ Al_2_O_3_ nanofluids. Larger values of modified Hartmann number lead to decay the temperature of γ Al_2_O_3_ nanofluids.

**Fig. 7:**
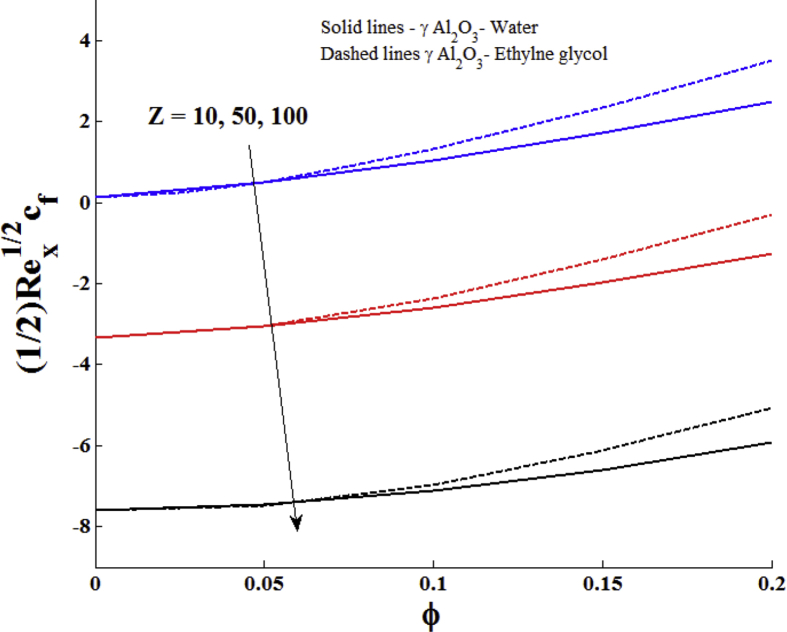
Impact of nanoparticle volume fraction and modified Hartmann number on local skin friction coefficient.

**Fig. 8:**
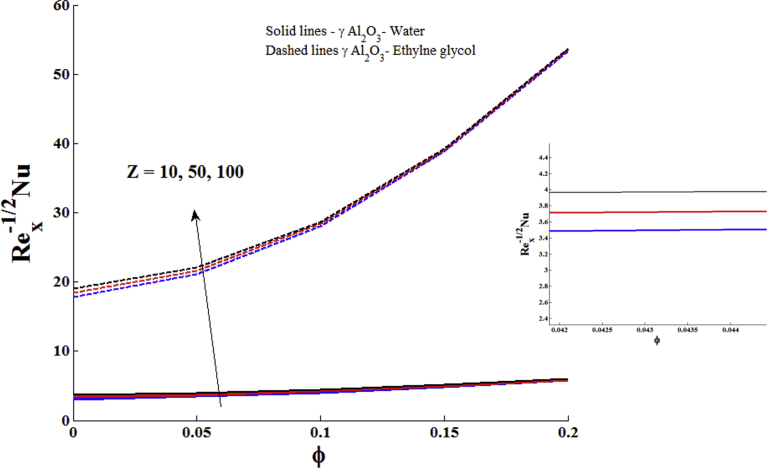
Impact of nanoparticle volume fraction and modified Hartmann number reduced Nusselt number with Pr = 6.96 (γ Al_2_O_3_- Water) and Pr = 204 (γ Al_2_O_3_- Ethylene glycol).

The impacts of Z and the ϕ on the skin friction coefficient and reduced Nusselt number are displayed in Figs. 7 and 8. It is seen that both skin friction and reduced Nusselt number are the increasing function of ϕ of γ Al_2_O_3_ nanofluids. The larger values of modified Hartmann number reduce the skin friction and increase the reduced Nusselt number.

## 4 Conclusion

Thermal transfer characteristics of steady state electro -MHD flow of γ Al_2_O_3_-Water/Ethylene glycol nanofluid over a stretchable Riga plate in two dimensional case are studied numerically. An effective Prandtl number model is used to analyse velocity and thermal boundary layers. The main results are summarized as follow:

• Higher nanoparticle volume fraction increases the velocity profile and decreases the temperature profile in γ Al_2_O_3_ nanofluids.
• The γ Al_2_O_3_- Ethylene glycol has larger velocity and lower temperature than γ Al_2_O_3_- Water.
• The velocity distribution increases with higher modified Hartmann number due to external electric field. The higher values of modified Hartmann number reduce the temperature profile.
• The local skin friction and reduced Nusselt number are the decreasing function of modified Hartmann number.

## Declarations

### Author contribution statement

N. Vishnu Ganesh, Qasem M. Al-Mdallal: Conceived and designed the analysis; Analyzed and interpreted the data; Contributed analysis tools or data; Wrote the paper.

Sara Al Fahel, Shymaa Dadoa: Analyzed and interpreted the data; Wrote the paper.

### Funding statement

This work was supported by UAE University.

### Competing interest statement

The authors declare no conflict of interest.

### Additional information

No additional information is available for this paper.
